# The Threshold and Lag Effects of Temperature on Pine Wilt Disease Show Significant Spatial Heterogeneity

**DOI:** 10.3390/insects16080834

**Published:** 2025-08-12

**Authors:** Ruicong Zhang, Jixia Huang, Xiaoting Zhao, Yanqing Liu, Guofei Fang, Yantao Zhou, Maogui Hu

**Affiliations:** 1 Precision Forestry Laboratory of Beijing, Beijing Forestry University, Beijing 100083, China; zhangruicong1823@igsnrr.ac.cn (R.Z.); xtzhao2025@163.com (X.Z.); yanqing666@bjfu.edu.cn (Y.L.); 2 State Key Laboratory of Resources and Environmental Information System, Institute of Geographical Sciences and Natural Resources Research, Chinese Academy of Sciences, Beijing 100101, China; humg@lreis.ac.cn; 3 Center for Biological Disaster Prevention and Control, National Forestry and Grassland Administration, Shenyang 110034, China; fgfly@163.com (G.F.); ytzhouly@163.com (Y.Z.)

**Keywords:** pine wilt disease, threshold effect, lag effect, DLNM

## Abstract

Pine wilt disease (PWD) is an economically important disease. With the increasing temperature caused by climate change, there is a concern that it may expand to regions currently at low risk, cause more serious ecological harm and economic losses in China. The pinewood nematode has an optimal temperature range for development, and historical meteorological conditions, particularly temperature, can influence its current occurrence through time-lagged effects. Lag effect is a temporally delayed effect. In order to investigate the threshold effect and lag effect of temperature on PWD, we collected province-level occurrence data in China and explored the threshold effect, lag effect, and spatial heterogeneity mechanism using a distributed lag non-linear model. The results show that temperature has a significant threshold effect and lag effect on PWD. The threshold temperatures and lag periods vary from 19.5 °C to 25.1 °C and from 1 to 3 months in different provinces in the study area. The threshold temperature shows significant spatial heterogeneity and is positively correlated with the average temperature. This study provides a theoretical basis for the prevention and control of PWD, as well as protection of forest ecological security.

## Simple Summary

As an economically important disease, pine wilt disease has posed severe threats to forest ecosystems both domestically and globally. Under the context of global warming, scientifically understanding the impact of temperature on pine wilt disease is critical for enhancing predictive and management capabilities. In this study, we investigate the thermal adaptation range of pinewood nematode under hot temperatures and elucidate its time-lag effects in response to temperature fluctuations within optimal thermal conditions. The threshold temperatures and lag periods vary from 19.5 °C to 25.1 °C and from 1 to 3 months in different provinces. Collectively, our findings establish a technical framework for analyzing threshold and lag effects in pine wilt disease, providing theoretical insights to support evidence-based prevention and control strategies.

## 1. Introduction

Pine wilt disease (PWD) is a globally significant forest disease [1,2], responsible for severe ecological and economic losses, particularly in East Asia, including China. The first occurrence of PWD was documented in 1982 at the Mausoleum of Dr. Sun Yat-sen in Nanjing, Jiangsu [3]. It quickly spread to surrounding areas following a pattern of “spread–stabilization–outbreak–full outbreak” [4]. Then, subsequent findings of PWD include Ganzhou, Jiangxi in 2003, Zibo, Shandong in 2013 [5], and northeastern China in 2016 [3], spreading from south to north. The disease spread rapidly across the country, especially in the eastern and southern regions, causing significant ecological harm and economic losses [4]. By the end of 2022, there were a total of 701 county-level epidemic areas nationwide (The National Forestry and Grassland Administration, 2022), indicating that pine wilt disease remains a significant threat in China. Although some progress has been made in controlling the disease, such as a reduction in the number of epidemic areas compared to 2020, the widespread distribution of the disease highlights the need for further research.

Every year, starting in April, the pinewood nematodes (PWNs, *Bursaphelenchus xylophilus*) congregate in the nymphal chambers of their vector beetles, infecting the host trees. By the time temperatures drop in October, PWNs begin to overwinter and the development of symptoms of PWD attenuates. Previous studies have established that the onset of PWD occurs from April to October [6,7,8]. As an invasive species, the PWN can infect dozens of pine tree species [9]. In China’s natural environments, pine species susceptible to PWN invasion include subtropical and tropical varieties such as Masson pine and Simao pine [10]. Infected pine trees die in a few months or even in a few weeks [11], and the death rate in China is as high as 100% [12]. After the pine trees die, the carbon and nitrogen contents decline, and ecological functions and wildlife habitats are damaged, which affect the forest ecological cycle [13]. Furthermore, against the backdrop of global warming, the suitable habitat for PWNs is gradually expanding. Global land surface temperature will rise by 0.3–0.7 °C from 2016 to 2035 [14]. Global warming helps spread PWNs and insect vectors (e.g., *Monochamus alternatus*), inhibits the growth of pine trees, and complicates the expression of diseases. PWD is principally found in warm areas, yet with increasing temperatures, it is expected to spread to other regions now at low risk [15,16,17]. The suitable area for PWNs in China will consistently expand [18,19]. Thus, PWD is a serious forest disease all over the world, especially in China, with a vast land source and various climatic zones.

As a typical poikilotherm, the PWN is sensitive to temperature [20]. Temperature affects the nematodes, insect vectors, and their host in various ways, including the development and reproduction of the PWN, the longevity of insect vectors, and the habitat suitability of pine trees [21]. PWNs begin to develop at 10 °C, and symptoms of PWD develop at an average temperature of 20 °C or more in Japan [22]. However, “the most suitable” temperature shows spatial heterogeneity. For instance, PWNs struggle to develop below 10 °C but thrive in the 10–25 °C range in the Yangtze River basin [23]. PWNs develop above 20 °C in New Jersey, with maximum growth rates occurring at 28-29 °C [24]. In the active period, high temperature and low rainfall may exacerbate the symptoms and quicken the spread of PWD [6]. Also, for insect vectors, high windspeed helps them and PWNs spread over long distances [23]. Good sunshine conditions promote the movement of PWNs within vectors and helps the further development of PWD [25]. High temperature also enhanced the susceptibility of the host trees [26]. In different areas, the sensitivity and response of PWNs to the same temperature are not exactly the same, showing spatial heterogeneity, but in general, they follow an inverted “U-shaped” relationship that first increases and then decreases with the increase in temperature; that is, there will be a “threshold” at a certain node.

The distributed lag non-linear model (DLNM) [27] uses a cross-basis function to simultaneously capture non-linear exposure–response and lag–response relationships in time series data. The DLNM has been widely applied in environmental science and public health to study the lag effects of meteorological factors on phenomena such as air pollution [28] and human diseases [29,30,31,32,33]. Lag represents the time interval between the exposure event and the outcome when evaluating the delay of the effect [27]. In forestry, the DLNM has also been used to explore the lag effects of climate conditions on tree growth [34]. Similarly, we applied the DLNM to investigate the potential lag effects of temperature on PWD, hypothesizing that historical meteorological conditions may influence current disease symptoms. Additionally, we used the Generalized Additive Model (GAM) [35] to fit non-linear relationships, as its smooth functions effectively handle multiple independent variables without increasing estimation variance. Together, these tools allow us to study the threshold and lag effects of temperature on PWD incidence.

Current studies on the effect of temperature on PWD primarily focus on two aspects: determining the suitable temperature range for the development and reproduction of the PWN and investigating the non-linear relationship between temperature and PWD. However, there is a noticeable research gap concerning the threshold effect and lag effect of temperature on PWD. In this study, we employed the DLNM to study the threshold effect and lag effect of temperature on PWD and explore spatial heterogeneity at the provincial level in China. We aimed to answer the following research questions: (1) Is there a threshold effect of temperature on PWD, and, if so, what is the threshold temperature? (2) Does a lag effect of temperature on PWD exist near the threshold temperature, and, if yes, what is the duration of the lag period? (3) Does spatial heterogeneity play a role in the threshold effect of temperature on PWD?

## 2. Materials and Methods

### 2.1. Study Area

**Figure 1:**
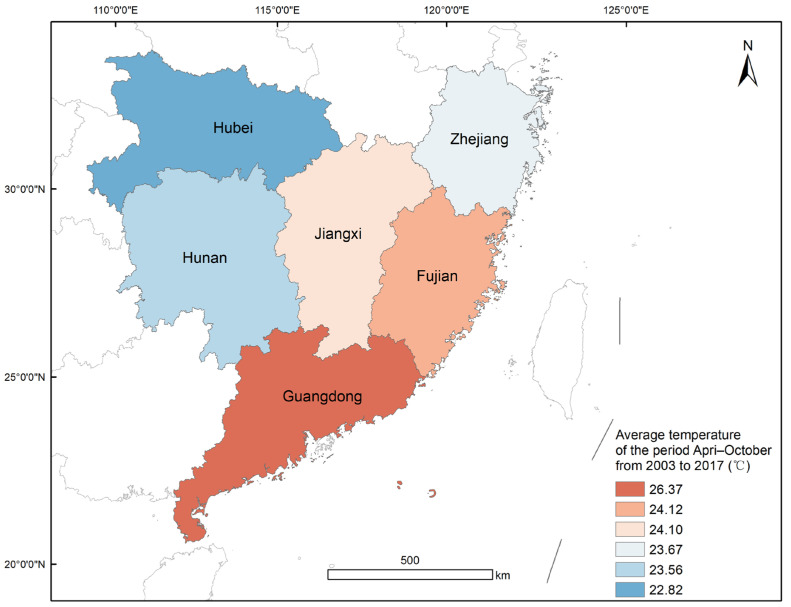
Study area with average temperatures for April–October from 2003 to 2017.

In this study, six provinces in southern China, Hubei, Zhejiang, Hunan, Jiangxi, Fujian, and Guangdong, were selected (Figure 1). The latitude and longitude range from 20.21° N to 33.28° N and 108.36° E to 122.95° E. The study area is bordered by the East China Sea to the east and the South China Sea to the south, encompassing both tropical and subtropical zones. The region exhibits diverse topographical features, characterized by a high north-west and low south-east topography, along with a central plain area accompanied by a basin area.

### 2.2. Data

We use incidence area, normalized difference vegetation index (NDVI), and average temperature between April and October from 2003 to 2017 in our study. Previous studies have established that the onset of PWD occurs from April to October [6]. The detailed data sources and calculating processes are introduced as follows.

Monthly incidence was calculated as the ratio of monthly incidence area to host area in the current year. We obtained the monthly incidence area from the survey data of the Biohazard Prevention and Control Center, National Forestry and Grassland Administration (NFGA) of the People’s Republic of China (https://www.forestry.gov.cn/fkzx.html, accessed on 1 January 2021). The data covers the period from 2003 to 2017 and includes information on PWD incidence between April and October each year. Our analysis utilizes the attribute of incidence area. In China, county-level forestry stations conduct annual surveys of PWD using ground patrols and aerial remote sensing. These surveys involve sampling, isolation, identification, confirmation, and detailed investigations. The forestry stations then report the statistical results, including the occurrence area and other relevant information within the district, to the Biohazard Prevention and Control Center of the NFGA for verification.

We calculated the host area using NDVI data. NDVI is one of the most commonly used remote sensing indexes for vegetation research. Long-term NDVI data is of great significance to the study of vegetation change. The calculation of NDVI is as follows:

(1) $$\begin{array}{c} \mathrm{NDVI}=\frac{NIR-RED}{NIR+RED} \end{array}$$

where, NIR represents the near-infrared band of the remote sensing image, while RED denotes the red band of the remote sensing image.

This study utilizes NDVI data obtained from the Chinese Academy of Sciences, Resources and Environment Science Data Center data registration and publication system (http://www.resdc.cn/DOI, accessed on 1 March 2021) to extract the area of host trees affected by the PWN. To eliminate the influence of other vegetation types such as broadleaf forests, the area of coniferous forests was extracted as the yearly host area of PWD using NDVI data for December each year from 2003 to 2017 according to previous literature [36]. We use an NDVI value greater than 0.2 as the criterion for extracting coniferous forests [37,38]. The use of NDVI data offers two key advantages. Firstly, it provides continuous temporal coverage, allowing for accurate monthly analysis. In contrast, the NFGA publishes a five-year period for the continuous forest inventory (CFI) in China and the GLC_FCS30 product data [39], while the NDVI data is accurate to the month. Secondly, NDVI data offers wide spatial coverage, which is more suitable. The GLC_FCS30 product data is missing in Guangdong Province and the ESACCI product data [40] differs significantly from the CFI data in Hubei Province and Zhejiang Province. The NDVI data covers the entire country.

Meteorological data from April to October each year between 2003 and 2017 were obtained. The daily meteorological data is sourced from the China Meteorological Data Center (http://data.cma.cn/, accessed on 1 March 2021) and includes information reported by 118 meteorological stations within the study area. The dataset comprises various elements, such as average temperature, precipitation from 20:00 to 20:00 (+1), average relative humidity, average 2 min wind speed, and sunshine duration; we use the average temperature in the formal analysis of following sections. First, outliers exceeding two times the standard deviation from the mean were removed from the dataset to ensure data quality. Furthermore, the meteorological parameters for each province were formed through interpolation to supplement missing values by second-order polynomial fitting, and the monthly average value was calculated. The monthly average temperatures of every province in our study area were formed, including Hubei, Zhejiang, Hunan, Jiangxi, Fujian, and Guangdong.

### 2.3. Distributed Lag Non-Linear Model

We employed DLNM models to explore the threshold effect and lag effect of temperature on PWD using the ‘dlnm’ package version 2.5.7 in R version 4.0.0 and used the Akaike information criterion (AIC) to test model fit. The modeling and fitting were employed on every province separately. The threshold effect refers to a turning point in the non-linear relationship between temperature and PWD incidence, where the trend of the curve on either side of the turning point differs or even reverses. The lag effect refers to the temporal delay in which temperature influences the PWD incidence, indicating that the current PWD incidence is affected not only by the current temperature but also by historical temperatures. The lag period is measured in months.

First, we used the GAM to fit the non-linear relationship between temperature and PWD incidence, which lays a foundation for the subsequent threshold effect research:

(2) $$\begin{array}{c} g\left(\mu_{Y}\right)=s_{0}+\sum_{j=1}^{n}s_{j}\left(x_{j}\right) \end{array}$$

where $\mu_{Y}$ means PWD incidence as the dependent variable, $x_{j}$ denotes different independent variables, we chose average temperature as the independent variable, $\sum_{j=1}^{n}s_{j}\left(x_{j}\right)$ refers to the function associated with the independent variable, which is called the basis function, we chose the spline function here to fit the non-linear relationship between average temperature and incidence, and $s_{0}$ is the residual.

Second, we conducted an analysis of the spatiotemporal effect of temperature on PWD using the DLNM. This approach allows us to investigate the threshold effect, lag effect, and spatial heterogeneity separately. The DLNM involves two basis transformations, which are utilized to construct the cross-basis. This cross-basis captures the exposure–response and lag–response relationships.

To assess the threshold effect and lag effect of temperature on PWD, we employed the DLNM:

(3) $$\begin{array}{c} g\left(Y_{t}\right)=\alpha +\sum_{j=1}^{J}s_{j}\left(x_{tj};\beta_{j}\right)+\sum_{k=1}^{K}\gamma_{k}u_{tk} \end{array}$$

where g() denotes link functions, $Y_{t}\left(t=1,2,\ldots ,n\right)$ represents PWD incidence as the dependent variable, $x_{j}\left(j=1,2,\ldots ,J\right)$ is the average temperature as the independent variable, $\sum_{j=1}^{J}s_{j}\left(x_{tj};\beta_{j}\right)$ refers to the cross-basis function, including basis functions of the dependent variable and independent variable and the independent variable and lag period, and $\beta_{j}$ represents the regression coefficient corresponding to the independent variable $x_{tj}$. $\sum_{k=1}^{K}\gamma_{k}u_{tk}$ denotes the linear effect of other confounding factors, for instance time series and meteorological elements such as precipitation, wind speed, and sunshine duration, $\gamma_{k}$ denotes the coefficient of the confounding factor $u_{tk}$, and α represents the intercept term.

We tried low, bilateral, and high threshold functions as the basis function between the independent and dependent variables, according to the range of suitable temperatures for PWD, the average temperature of the study area, and the modeling results; we employed the high threshold type and the natural cubic spline to transform the non-linear relationship and the lag effect, respectively. The AIC evaluates model fitting superiority; the model achieves the best performance when the AIC is at its minimum. We calculated AIC values for each 0.1 °C of the DLNM and obtained the results of threshold temperature. We assessed the relationship between cumulative relative risk (${RR}_{cum}$) and lag period to determine the lag period. Relative risk (RR) is the ratio of the risk at a specific temperature to the disease risk at the reference temperature:

$$RR\left(x,L\right)=\frac{\lambda (x_{j},L)}{\lambda (x_{ref})}$$

where $\lambda \left(x_{j},L\right)=exp(\alpha +s_{j}\left(x_{tj};\beta_{j}\right)+\gamma_{k}u_{tk})$ is the expected PWD incidence, $s_{j}\left(x_{tj};\beta_{j}\right)$ is the cross-basis function, $\gamma_{k}u_{tk}$ represents the combined effect of other confounding factors, α represents the intercept term, and $\lambda (x_{ref})$ denotes the expected incidence at the reference temperature.

We tried from 4 to 12 months for the maximum lag period; the results of lag period are the same as the lag results below. Considering that the onset of PWD spans approximately 6–7 months from April to October [6,7], we ultimately set the maximum lag period as 6 months. The ${RR}_{cum}$ is defined as follows:

$${RR}_{cum}\left(x\right)=exp(\sum_{l=0}^{6}s_{j}\left(x_{tj},l;\beta_{j}\right)-s_{j}\left(x_{ref},l;\beta_{j}\right))$$

If the cumulative relative risk is consistently less than 1 when the “lag period” on the *X*-axis is less than n, it suggests that the lag period is n.

To explore the spatial heterogeneity, we used Spearman rank correlation to explore the relationship between threshold temperature and average, maximum, and minimum temperature in different provinces.

## 3. Results

### 3.1. PWD Spatiotemporal Pattern Evolution

**Figure 2:**
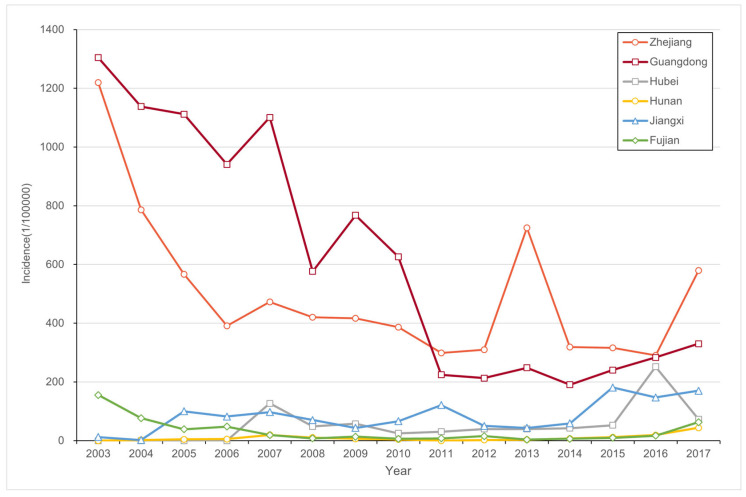
Time series of PWD incidence.

From 2003 to 2017, there was a notable pattern of higher incidence in Zhejiang and Guangdong Provinces and lower incidence in other provinces (Figure 2). In Hubei Province, PWD was initially detected in 2007, and the incidence remained relatively stable at around 0.1%. However, after 2015, there was a rapid increase in incidence, reaching levels of 0.1–0.3%. Zhejiang Province and Guangdong Province experienced relatively high PWD incidence, but there was a decreasing trend over time. In Zhejiang Province, the incidence decreased from over 2% in 2003, peaking again in 2013, and declining to around 1% by 2017. Similarly, in Guangdong Province, the incidence decreased from over 3% to around 0.5%. The situation in Hunan Province was less severe, with PWD detection in 2004 and a peak incidence of nearly 0.05% in 2007. After that, the incidence remained low until a resurgence occurred after 2015. In Jiangxi Province, there was a sharp rise in incidence to about 0.25% after 2006, followed by a decrease and subsequent increase after 2015. In Fujian Province, the incidence was high from 2003 to 2006, reaching a maximum of approximately 0.3%, but remained at a low level until 2017.

### 3.2. The Threshold Effects of Temperature on PWD

**Figure 3:**
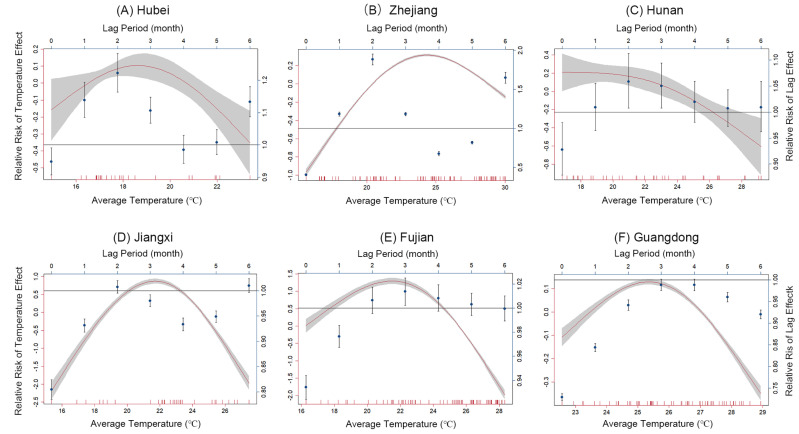
The estimated effects of temperature on incidence and the distributed lag periods of temperature-related incidence. (**A**) Hubei Province, (**B**) Zhejiang Province, (**C**) Hunan Province, (**D**) Jiangxi Province, (**E**) Fujian Province, and (**F**) Guangdong Province.

There was an inverted U-shaped relationship between temperature and the incidence of PWD (Figure 3). The red lines represent the relative impact of temperature on incidence with different lag periods, and the gray areas indicate the 95% confidence intervals. Below the threshold temperature, the incidence of PWD tended to increase or remain stable with rising temperatures, while it decreased above the threshold. The curve for Hunan Province showed a smaller slope compared to others. At lower temperatures to the left of the threshold, the curve remained stable. There was an obvious threshold effect of temperature on PWD, with threshold temperatures varying significantly across provinces. These threshold temperatures were 19.5 °C in Hubei Province, 21.0 °C in Hunan Province, 22.5 °C in Jiangxi Province, 22.7 °C in Fujian Province, 23.1 °C in Zhejiang Province, and 25.1 °C in Guangdong Province.

### 3.3. The Lag Effects of Temperature on the PWD

Temperature exhibited a lag effect on PWD (Figure 3). The blue data dots with error bars depict the relative risks of heat-related PWD incidence, with the error bars showing the range of values for the 95% confidence intervals. Across all provinces in the study area, the relative risk was below 1 when the lag was 0, indicating a negative influence of temperature on PWD. Our findings revealed a lag effect of 1 month in Hubei Province, Zhejiang Province, and Hunan Province, a 2-month lag effect in Jiangxi Province and Fujian Province, and a 3-month lag effect in Guangdong Province. This implied that not only does the temperature in the current month significantly influence PWD, but also the temperatures from the preceding 1 to 3 months have a notable lag effect on PWD.

### 3.4. Correlation of Threshold Effect, Lag Effects, and Temperature

**Figure 4:**
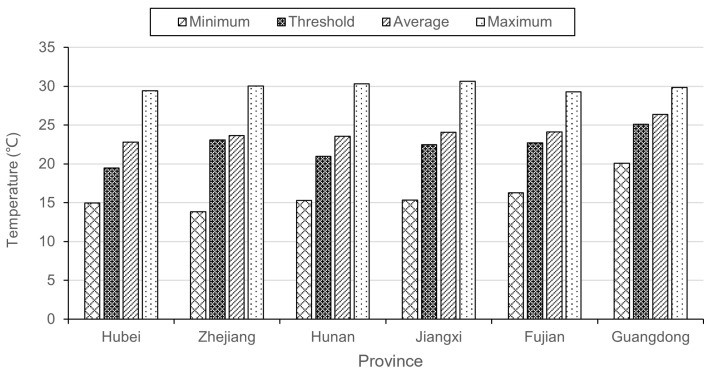
The contrast of threshold, average, maximum, and minimum temperature in different provinces.

The regional variation in threshold temperatures was found to be statistically significant. The variation in threshold temperatures across regions followed a similar pattern to that of average, maximum, and minimum temperatures. Specifically, regions with higher average temperatures, such as Guangdong Province, Zhejiang Province, and Fujian Province, tended to have higher threshold temperatures. This pattern was also observed in maximum and minimum temperatures (Figure 4). Spearman’s rank correlation analysis revealed a significant positive correlation between threshold temperature and average temperature (rho = 0.829). However, no significant correlations were found between threshold temperature and maximum temperature (rho = −0.086) or between threshold temperature and minimum temperature (rho = 0.429). The spatial heterogeneity in the threshold effect of temperature on PWD emphasized that as average temperatures increase, the corresponding threshold temperatures also tend to rise. For the lag effect, there was also a strong positive correlation (rho = 0.99) between the lag periods and threshold temperatures.

## 4. Discussion

This study utilized the DLNM to investigate the impacts of the average temperature on PWD incidence in some provinces of southern China. According to the results, there are threshold effects and lag effects in the influence of average temperature on PWD incidence. The correlation between average temperature and PWD incidence showed an inverted U-shaped curve, with the highest incidence at the threshold temperature. Temperature has a lag period of 1–3 months for PWD incidence, and the risk of PWD gradually increases after this period. PWD is not only affected by the immediate effect of current temperature, but it is also affected by the lagging historical air temperature. Through comparative analysis of the threshold effect in different provinces, we found significant spatial heterogeneity of the threshold effect of temperature on PWD incidence.

From 2003 to 2017, China’s overall PWD incidence exhibited a declining trend, attributed to nationwide epidemic area management and infected wood control measures (The National Forestry and Grassland Administration, 2018). However, significant variations existed among provinces. These differences likely stem from climatic factors and variations in PWN transmission. PWD was first detected in Nanjing, Jiangsu Province, before spreading to other regions [3,4]. As a poikilothermic organism, the PWN reproduces and spreads more rapidly under favorable temperatures [20].

Temperature has a threshold effect on PWD incidence. PWD is a poikilotherm organism, and its development and reproduction are highly influenced by temperature, especially high temperatures. Climate can affect the survival and development of PWD through both direct [24] and indirect influences on the host [41] and insect vectors [42,43]. Under high-temperature conditions, the protein expression in PWNs is significantly affected [44], the growth and development of PWNs are inhibited, and survival becomes difficult [20]. In the study area, the threshold temperature is between 19 and 25 °C, which gives similar results to previous studies [23]. When the temperature exceeds 30 °C, the developmental rate of PWN vector beetles decreases significantly, with a lethal upper threshold considered to be between 32 and 35 °C [45]. Also, it is hard for PWNs to develop at low temperatures. PWNs enter the third-stage dispersal juvenile (JIII) phase in winter, which is a growth-arrested stage. Temperature also limits the occurrence of PWD by affecting insect vectors. When the temperature drops below 0 °C, the survival rate of overwintering larvae of the vector *Monochamus* species significantly decreases [46]. However, high temperature thresholds are detected but low temperature thresholds are not in this study. This is due to the low latitude and warm climate of the study area.

Temperature exhibits a lag effect on PWD; historical temperatures exert a significant lag effect on PWD incidence, and the lag period is positively related to the temperature. Generally, high temperature induces stress in trees and exacerbates pest outbreaks and trees’ death [47,48]; high temperature increases the risk of infection by affecting trees and exacerbating PWNs, which may have an “incubation period”.

The threshold temperature for PWD exhibits significant regional variation and PWNs are local adaptable to the most suitable temperature [49]. Different studies have proposed different optimal temperatures for PWNs. In addition, it is shown that there is a positive correlation between threshold temperature and regional average temperature, which aligns with prior research [50]. A study in the middle and upper reaches of the Yangtze River found that 10–25 °C is suitable for the development of PWNs [23]. This region is mostly plateau and mountainous with a relatively low average temperature. However, PWNs in Ibaraki Prefecture, Japan, which belongs to a temperate oceanic monsoon climate with warm winters and cool summers, develop most rapidly in the range of 25–30 °C [22]. By contrast, in New Jersey, the environment above 20 °C is conducive to the development of PWNs, and the growth rate is the largest at 28–29 °C [24]. New Jersey is located in the temperate humid climate and subtropical monsoon humid climate zone, which has a high temperature and rain in summer and is warm and humid in winter. Several studies have biologically explained the adaptive characteristics and mechanisms of PWNs [51,52]. The PWN genome contains modifiable sites that alter gene expression in response to the environment, resulting in phenotypic adaptation. Furthermore, some adaptive traits are heritable, leading to population-level environmental adaptation among regional PWN populations [53].

Several limitations should be acknowledged in our study. Firstly, our analysis is conducted at the provincial level, whereas the PWN is an invasive pest that may exhibit varying frequencies in different prefecture-level cities. Additionally, microclimatic conditions within provinces may introduce variations that we could not account for in our analysis. Secondly, our study area encompasses six provinces in southeastern China, which, although relatively small, still exhibit some climatic variability. We acknowledge that these provinces share similar climatic factors, which is why we opted for provincial-scale analysis in this paper. However, future research may benefit from more fine-grained spatial analyses to capture localized variations in PWN incidence and its relationship with temperature.

This study explores the influence of average temperature on PWD and the threshold effect and lag effect. The relationship between temperature and PWD incidence is an inverted U-shaped curve. We find the threshold temperature and lag period in the relationship, with the threshold temperature exhibiting spatial heterogeneity linked to average temperature. The threshold temperatures and lag periods vary from 19.5 °C to 25.1 °C and from 1 to 3 months in different provinces. This study provides a theoretical foundation for PWD control and prevention, which is significant to forest ecological stability and sustainable forest management.

## Data Availability

The data presented in this study are available on request from the corresponding author due to privacy.

## Abbreviations

The following abbreviations are used in this manuscript:

PWD	Pine wilt disease
PWN	Pine wilt nematode, *Bursaphelenchus xylophilus*
DLNM	Distributed lag non-linear model
GAM	Linear dichroism Generalized Additive Model
